# Denonvilliers’ fascia acts as the fulcrum and hammock for continence after radical prostatectomy

**DOI:** 10.1186/s12894-021-00943-z

**Published:** 2021-12-17

**Authors:** Xuwei Lu, Chang He, Sihong Zhang, Fan Yang, Zhuifeng Guo, Jiaqi Huang, Minke He, Jiawen Wu, Xia Sheng, Wenyao Lin, Jie Cheng, Jianming Guo, Hang Wang

**Affiliations:** 1 Department of Urology, Minhang Hospital, Fudan University, Shanghai, 201199 China; 2 Department of Urology, Zhongshan Hospital, Fudan University, Fenglin Rd 130, Shanghai, 200032 China; 3 Department of Pathology, Minhang Hospital, Fudan University, Shanghai, 201199 China; 4 Department of Urology, Xuhui Hospital, Fudan University, Shanghai, 200031 China

**Keywords:** Prostate cancer, Prostatectomy, Denonvilliers’ fascia, Urinary continence, Positive surgical margin

## Abstract

**Background:**

Radical prostatectomy (RP) is the primary treatment of localized prostate cancer. Immediate urinary incontinence post-RP was still common and depressing without specific reason.

**Methods:**

A multicenter cohort of 154 consecutive patients from 2018 to 2020, who was diagnosed with localized prostate cancer underwent either modified mini-incision retropubic radical prostatectomy (Mmi-RRP) or laparoscopic radical prostatectomy (LRP) or robotic-assisted radical prostatectomy (RARP). Seventy-two patients with Denonvilliers’ fascia (DF) spared were included in DFS (Denonvilliers’ fascia sparing) group. Whereas eighty-two patients with DF completely or partially dissected were set as Group Control. The primary outcome was immediate continence (ImC). Continuous data and categorical data were analyzed with *t*-test and *Chi*-square test, respectively. Odds ratios (*ORs*) were calculated with logistic regression.

**Results:**

Urinary continence of Group DFS was significantly better than that of Group Control at each time point within one year after operation. Incidence rate of continence in Group DFS and Group Control were 83.3% vs 13.4% (*P* < 0.01) for ImC, 90.3% vs 30.5% (*P* < 0.01) at 3 months, 91.7% vs 64.6% (*P* < 0.01) at 6 months, and 93.1% vs 80.5% (*P* = 0.02) at 1 year after operation, respectively. Positive surgical margin (PSM) showed no significant difference (20.8% vs 20.7%, *P* = 1.0). In multivariate analysis, DFS showed importance for ImC post RP (*OR* = 26.4, *P* < 0.01).

**Conclusions:**

Denonvilliers’ fascia acted as the fulcrum and hammock for continence post RP. Preservation of DF contributed to better continence after RP without increase of PSM.

*Trail registration* Our research was conducted retrospectively and approved by the ethical committees of Minhang Hospital, but not registered.

## Introduction

Prostate cancer (PCa) is the top-ranking malignant tumor for men worldwide [1]. Radical prostatectomy (RP) is the primary therapeutic choice for localized PCa. Dramatic improvement of surgery technique and more application of newer devices have reduced the incidence of severe complications for RP. Unfortunately, incontinence after RP, which haunted patients extremely hard, is still a huge challenge. The morbidity of incontinence one-year after RP remains up to 21%, regardless whether open radical prostatectomy (ORP) or laparoscopic radical prostatectomy (LRP) or robotic-assisted radical prostatectomy (RARP) has been taken [2]. Although many researchers have proposed many factors that might matter such as sphincter, length of membrane urethra, bladder neck preservation, neurovascular bundle (NVB) sparing, and Denonvilliers’ fascia (DF) as well, that is still uncertainty about the mechanism of post RP continence [3].

Furthermore, most literatures reported continent rate of short-term, mid-term and long-term post RP. As to immediate continence (ImC), few reports could be found. We have applied a new technique as DF-sparing (DFS) since 2018 in RP with excellent urinary continence, especially the ImC. Because of the encouraging outcome of urinary control, there must be something worth thinking about. So, we analyzed the data to explore the mechanism of continence post RP and the role of DF in it, as well as the feasibility and safety of DFS.

## Methods

From January 2018 to October 2020, 154 consecutive patients with localized prostate cancer underwent RP in Zhongshan Hospital, Minhang Hospital and Xuhui Hospital, FUDAN University. All the patients were given bone scan or PET-CT and excluded with metastasis before operation. The majority of patients completed MRI before operation except for those with contraindications including metal implantation and claustrophobia. Any suspicious sign showing that prostate capsule was invaded whether in radiology or in surgery, especially at the lateral-posterior direction, DF sparing was not recommended. If tumor was localized and prostate capsule wasn’t invaded, DFS technique was available. Seventy-two cases that underwent either modified mini-incision retropubic radical prostatectomy (Mmi-RRP) or LRP or RARP with DFS were included in Group DFS. Group Control included 82 cases received Mmi-RRP/LRP/RARP with DF completely or partially dissected.

All the procedures were completed by three experienced surgeons. Due to puncture approach, local invasion or neoadjuvant therapies, DFS was applied to appropriate patients. Urinary catheter was removed 5–7 days after operation. All the patients were followed up for 2–26 (14.5 ± 6.9) months.

The primary outcome was ImC. All the continence status were assessed by outpatient follow-up. Continent status reported by patients was collected at 1-week, 3-month, 6-month, and 1-year after urethral catheter was discarded. We defined continence as usage of ≤ 1 pad per 24 h. Considering that urine leakage caused by multiple factors was prevalent shortly after catheter removal, most of which was transient and could recover soon without treatment, we defined ImC as regaining continence within 1-week after urinary catheter removal [4].

### Surgery and post-surgery protocol

**Fig. 1:**
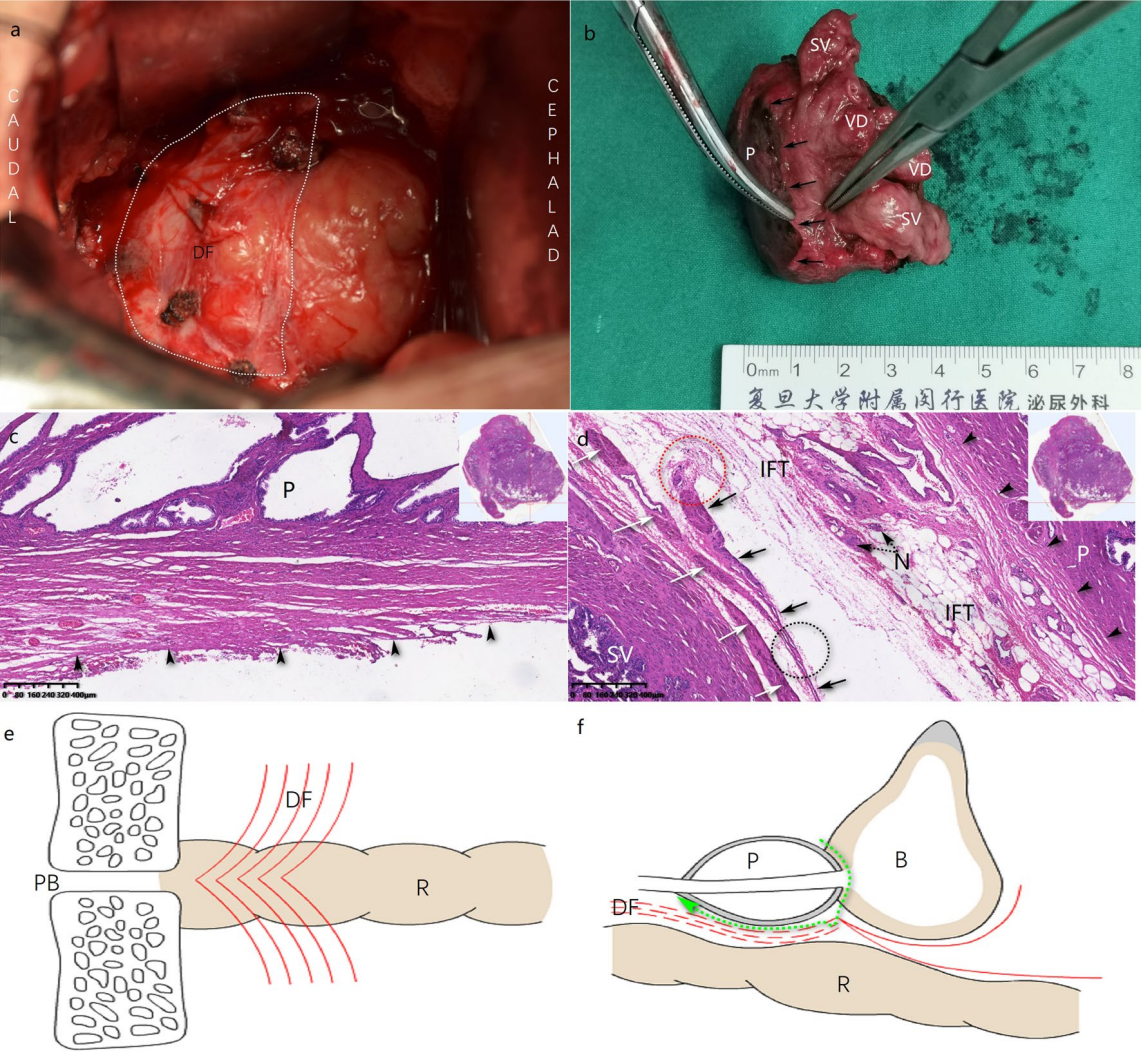
Scenes of Mmi-RRP procedure with DFS technique and pathological section through paramedian of prostate, stained by HE. **a** DF (white dot line area) kept intact after prostate removal, also shown on schematic drawing (**e**). **b** Anterior layer of DF cut open at seminal vesicles triangle level. Dissection was kept between DF and PC towards apex of prostate. Black arrows exhibited the cut-line of DF’s anterior layer. **c** No DF structure in the backward direction of prostate. **d** The cut-line of DF on the pathological section (red dot line circle), fused with seminal vesicle fascia propria (black dot line circle). **e**, **f** Schematic drawing OF dissecting bladder and prostate junction (green dot line). B: bladder; HE: hematoxylin and eosin; IFT: inter fascial tissue; N: nerve; P: prostate; PB: pubic bone; PC: prostate capsule; R: rectum; SV: seminal vesicles; VD: vas deferens. Black arrow: DF; Black arrow head: PC; White arrow: seminal vesicle fascia propria

**Fig. 2:**
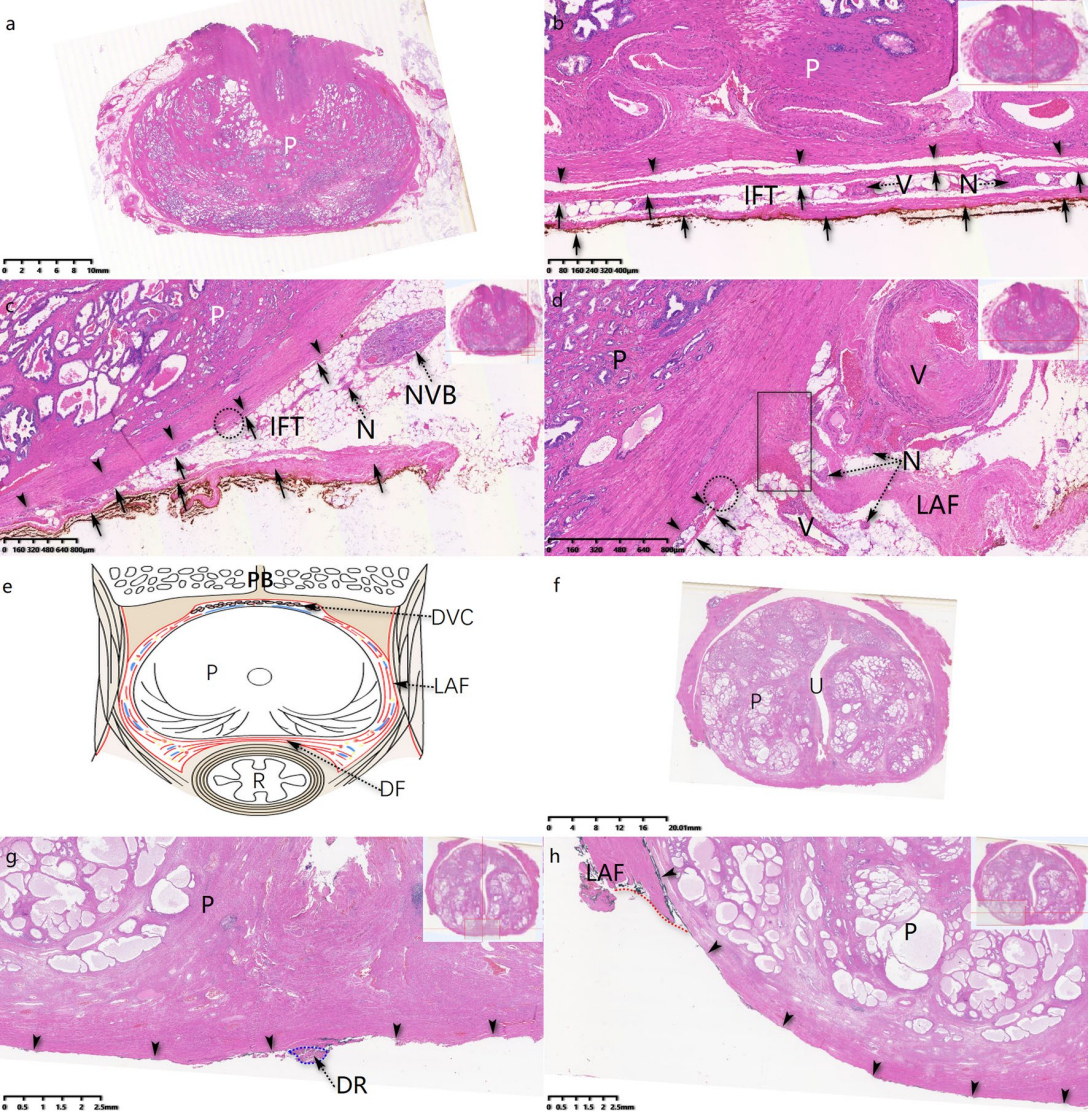
Transverse sections at mid-prostate in Group Control (**a**–**d**) and Group DFS (**f**–**h**), both stained by HE. **a** Overall vision of pathological section from Group Control. **b** In 6-o’clock direction of prostate, two layers of DF were dissected with prostate. **c** In 4-o’clock direction, anterior layer of DF extended anterolaterally alongside with prostate capsule, sometime fused together (show in black dot line circle). Middle layer of DF and NVB were removed with prostate. **d** Anterior layer of DF extended anterolaterally and connected with levator ani fascia (LAF) at 3-o’clock direction. **e** Schematic drawing simulated peri-prostate fascia structure including DF and LAF. **f** Overall vision of pathological section from Group DFS. **g** In 6-o’clock direction of prostate, few DR fragments without DF layers. **h** In 8-o’clock direction, DF was spared successfully with no DF structure visible in the specimen. DR: dorsal raphe; DVC: dorsal vascular complex; IFT: inter fascial tissue; LAF: levator ani fascia; N: nerve; P: prostate; PB: pubic bone; R: rectum; V: vessel. Black arrow: DF; Black arrow head: PC; Black rectangle: anterior layer of DF adhered with LAF; Red dot line: cut line of LAF

All the procedures were operated out of peritoneal under general anesthesia. The surgical approaches were decided by financial conditions and personal preferences of patients with full knowledge of benefits and risks. Mmi-RRP was operated with an approximate 7 cm longitudinal suprapubic incision, whereas LRP or RARP was with 3–4 poles. DF was thought to be composed of multiple layers and ensheathed both seminal vesicles and vas deferens into the anterior layer. After isolation of seminal vesicles, anterior layer of DF was isolated at the level of seminal vesicle triangle, and the dissection was located between prostate capsule (PC) and DF towards prostate apex in order to keep DF intact (Figure 1, Figure 2f–h). For the sake of cavernous nerves protection, blunt dissection was preferred. In consideration of potential nerve fiber damage, energy devices were seldom used during dissection near prostate.

Unfortunately, it was difficult to keep DF intact sometimes. In locally advanced PCa cases, the tight junction between DF and prostate added extra difficulty to LRP procedures. In these situations, DF would be resected partially or completely together with prostate (Fig. 2a–d). As to NVB sparing, careful dissection along PC in post-lateral direction should be enforced in some suitable cases.

No bladder neck and anterior and/or posterior reconstruction were conducted in our procedures. It was necessary to dissect close to prostate and keep bladder neck intact. But some situations, for example, locally advanced PCa, big prostate volume, and irregularly shaped prostate could make it hard to keep bladder neck intact. Under these circumstances, reconstruction of bladder neck was made to restore anastomosis, always by suturing bladder neck at 6 o’clock position.

### Statistical analysis

The pathology images were processed with K-viewer version 1.5.3.1 (KFBIO, Zhejiang, CHINA). The continuous data and categorical data were analyzed with *t*-test and *Chi*-square test, respectively. Kaplan–Meier method was used to compute urinary continence curve. Binary logistic regression analysis was applied to screen predictors for ImC. All data analyses were performed using SPSS 22.0 statistical software (IBM SPSS, Chicago, IL, USA). In all analysis, *P* < 0.05 was considered statistically significant.

## Results

**Fig. 3:**
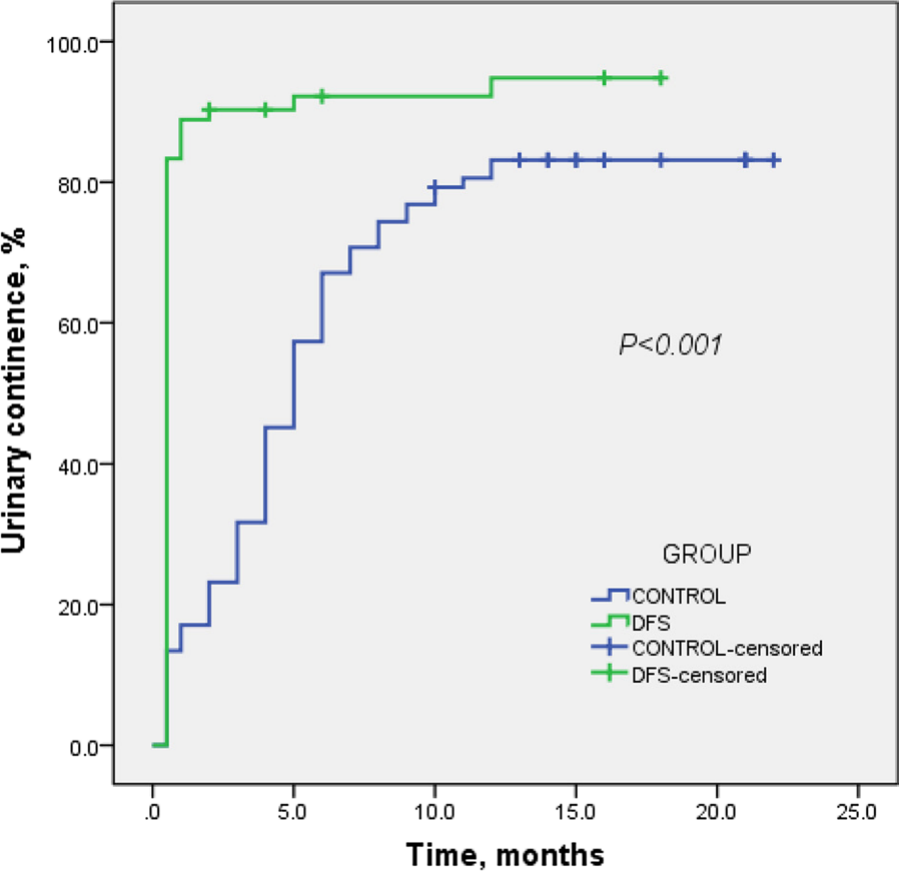
Kaplan–Meier analysis for time to urinary continence post-surgery

All the 154 consecutive patients completed RRP successfully and were followed-up for (14.5 ± 6.9) months, ranging from 2 to 26 months. The demographic and tumor characteristics of patients were shown in Table 1, while the details of procedures and pathology in Table 2. There was shorter duration of surgery (*P* < 0.01), more NVB sparing (*P* < 0.01), and less seminal vesicle invasion (*P* = 0.04) in Group DFS vs Group Control. Especially, the post-surgery continence condition was better in Group DFS than in Group Control at each time point from ImC to 3 months, 6 months (*P* < 0.01) and 12 months (*P* = 0.02), showed in Fig. 3.

**Table 1:** Demographics and tumor characteristics of patients

	Group DFS (n = 72)	Group Control (n = 82)	*P*
Mean ± Std (Range)			
Age, years	67.8 ± 5.0 (58–79)	69.3 ± 6.5 (54–80)	0.1
BMI	24.5 ± 2.6 (17.4–30.9)	24.5 ± 2.7 (16.8–30.3)	0.9
Diagnosis PSA, ng/ml	16.0 ± 12.5 (0.4–61.0)	38.9 ± 114.7 (4.1–946.6)	0.1
Prostate volume, ml	49.2 ± 15.8 (13.5–100.7)	51.5 ± 18.5 (16.7–122.5)	0.4
Clinical stage, n (%)			0.8
T1	30 (41.7)	33 (40.2)	
T2	38 (52.8)	46 (56.1)	
T3	4 (5.6)	3 (3.7)	
Diagnosis Gleason, n (%)			0.2
≤ 6	31 (43.1)	26 (31.7)	
7	31 (43.1)	37 (45.1)	
≥ 8	10 (13.9)	19 (23.2)	
Diagnosis method, n (%)			0.5
Biopsy	66 (91.7)	78 (95.1)	
TURP	6 (8.3)	4 (4.9)	
IIEF-5 Q_2_ ≥ 3 pre-surgery, n (%)	49 (68.1)	47 (57.3)	0.2
Comorbid condition, n (%)			
CAD	9 (12.5)	10 (12.2)	1.0
DM	12 (16.7)	17 (20.7)	0.5
HBP	38 (52.8)	39 (47.6)	0.5
COPD	8 (11.1)	4 (4.9)	0.2
CVD	4 (5.6)	6 (7.3)	0.8

*BMI* body mass index, *CAD* coronary artery disease, *COPD* chronic obstruction pulmonary diseases, *CVD* cerebrovascular diseases, *DM* diabetes mellitus, *HBP* high blood pressure, *TURP* transurethral resection of prostate

IIEF-5 Q2: International Index of Erectile Function-5, question NO.2. ‘‘When you had erections with sexual stimulation, how often was your erection hard enough for penetration during the last 3 months?’’ The following responses were available: ‘‘No sexual activity’’ (0); ‘‘Almost never or never’’ (1); ‘‘A few times (much less than half the time)’’ (2); ‘‘Sometimes (about half the time)’’ (3); ‘‘Most times (much more than half the time)’’ (4); and ‘‘Almost always or always’’ (5)

**Table 2:** Outcomes of surgery

Group	DFS, n (%)	Control, n (%)	*P*
Mean ± Std (Range)			
Surgery duration, min	153.2 ± 66.4 (100–400)	188.3 ± 65.1 (90–480)	< 0.01
Blood loss, ml	143.1 ± 107.2 (20–800)	132.4 ± 136.9 (20–1000)	0.6
NVB sparing			< 0.01
Bilateral	27 (37.5)	24 (29.3)	
Unilateral	13 (18.1)	2 (2.4)	
None	32 (44.4)	56 (68.3)	
LND	63 (87.5)	74 (90.2)	0.6
pT			0.5
T0	1 (1.4)	0 (0)	
T2	53 (73.6)	57 (69.5)	
T3	18 (25.0)	25 (30.5)	
Gleason scores			0.1
0	1 (1.4)	0 (0)	
≤ 6	17 (23.6)	9 (11)	
7	38 (52.8)	50 (61)	
≥ 8	16 (22.2)	23 (28)	
Positive LN	0 (0)	3 (3.7)	0.2
SV invasion	7 (9.7)	18 (22)	0.04
PSM	15 (20.8)	17 (20.7)	1.0
Sites of PSM			0.3
One-site (*location; %*)	13 (*5 basal, 7 apical, 1 post-lateral; 18%*)	11 (*2 basal, 8 apical, 1 post-lateral; 13.4%*)	
Multiple-sites (*locations; %*)	2 (*2 apical and post-lateral; 2.8%*)	6 (*5 apical and basal, 1 apical, basal and post-lateral; 7.3%*)	
Post-lateral PSM	3 (4.2%)	2 (2.4%)	0.7
IIEF-5 Q_2_ ≥ 3 post-surgery	25 (34.7)	14 (17.1)	0.01
Continence			< 0.01
Immediate	60 (83.3)	11 (13.4)	< 0.01
3 months-	65 (90.3)	25 (30.5)	< 0.01
6 months-	66 (91.7)	53 (64.6)	< 0.01
12 months-	67 (93.1)	66 (80.5)	0.02

*LND* lymph node dissection, *NVB* nerve vascular bundle, *PSM* positive surgical margin, *SV* seminal vesicles

In univariate analysis, surgery type (*OR* = 1.2–13.0, *P* < 0.01), surgery duration (*OR* = 1.0, *P* < 0.01), IIEF-5 Q2 pre-surgery (*OR* = 1.4, *P* = 0.03), Gleason score (*OR* = 0.7, *P* = 0.02), SV invasion (*OR* = 0.2, *P* < 0.01), NVB sparing (OR = 2.1–4.8, *P* = 0.01) and DF sparing (*OR* = 32.3, *P* < 0.01) were significantly related with ImC. While in multivariate analysis, only DF sparing was proved to be statistically significant for ImC, *OR* = 26.4, *P* < 0.01 (Table 3).

**Table 3:** Logistic regression analysis for predictors of immediate continence

ImC	Univariate		Multi-variate	
	*OR* (95%CI)	*P*	*OR* (95%CI)	*P*
Surgery type		< 0.01		0.6
Mmi-RRP *vs* LRP	13.0 (5.7–29.7)	< 0.01	1.9 (0.4–8.7)	0.4
RARP *vs* LRP	1.2 (0.4–4.0)	0.7	0.8 (0.2–3.6)	0.7
LND, Yes *vs* No	0.7 (0.3–2.0)	0.6		
Gleason score	0.7 (0.5–0.9)	0.02	0.9 (0.5–1.6)	0.8
SV invasion, Yes *vs* No	0.2 (0.1–0.7)	< 0.01	0.3 (0.1–1.3)	0.1
PSM, Yes *vs* No	0.9 (0.4–1.9)	0.8		
TURP before, Yes *vs* No	1.2 (0.3–4.3)	0.8		
Diagnosis PSA	1.0 (0.992–1.003)	0.4		
IIEF-5 Q_2_ pre-surgery	1.4 (1.0–1.9)	0.03	1.6 (0.9–2.7)	0.1
Prostate volume	1.0 (0.98–1.01)	0.6		
Age	1.0 (0.91–1.02)	0.2	1.0 (0.9–1.1)	0.6
BMI	0.9 (0.8–1.1)	0.3		
Surgery duration	1.0 (0.98–0.99)	< 0.01	1.0 (0.981–1.002)	0.1
Blood loss	1.0 (0.996–1.001)	0.3		
NVB sparing		0.01		0.9
Unilateral vs No	4.8 (1.4–16.4)	0.01	0.8 (0.1–4.3)	0.8
Bilateral vs No	2.1 (1.1–4.3)	0.04	1.1 (0.3–3.8)	0.9
DF sparing, Yes *vs* No	32.3 (13.3–78.4)	< 0.01	26.4 (8.4–83. 3)	< 0.01

*BMI* body mass index, *CI* confidence interval, *ImC* immediate continence, *LND* lymph node dissection, *NVB* nerve vascular bundle, *OR* odds ratio, *PSM* positive surgical margin, *SV* seminal vesicles

As to definition of sexual dysfunction, we used cutoff value between response 2 and 3 for question NO.2 in IIEF-5 [2]. There were more potent patients in Group DFS than in Group Control at 12 months post-surgery, (34.7% *vs* 17.1%, *P* = 0.01). No statistical difference of PSM was found between two groups (20.8% *vs* 20.7%, *P* = 1.0, Table 2).

## Discussion

Tumor control, urinary continence and sexual potency make up the Trifecta of RP. Post-surgery incontinence is one of the most functionally devastating complications, which could make patients too depressed to maintain social relationship and can even lead to suicide. Albkri A et al. [5] figured out that 28.1% patients regretted for RP decision because of complications. Unfortunately, there is so far no universal definition of continence up to now, which makes the morbidity of continence post-RP vary [6]. As literatures reported, ImC varied from 18 [7] to 63.5% [8], while continence at 3 months from 45 [9] to 82% [10], and continence at 12 months from 79.8 [2] to 91% [11]. In consideration of urinary consequences on record, morbidity of incontinence after RP in real world could probably be higher than reported [12]. Grivas N et al. [13] pointed out that continence at 12 months post-surgery was only 49.2% with strict definition.

The debate between cancer free and continence still lacks conclusion. Routinely, the more tissue near the tumor incised, the better tumor control. But it also means more potential functional structures to be cut, and incontinence is more likely to happen. Therefore, the discovery of landmark structure for urinary continence is needed. There are many hypotheses, such as membrane urethra length, urethral sphincter complex, detrusor apron of bladder neck, pelvic floor musculature, DVC, and NVB [3], etc. Asimakopoulos AD et al. [14] preferred complete periprostatic anatomy preservation to protect maximal functional structure and got 100% continence (≤ 1 pad per day) just at the time of catheter removal. However, this procedure may not be suitable for patients with higher tumor stage. Here we suppose DF to be the answer, particularly for the early-term and mid-term urinary control after RP.

**Fig. 4:**
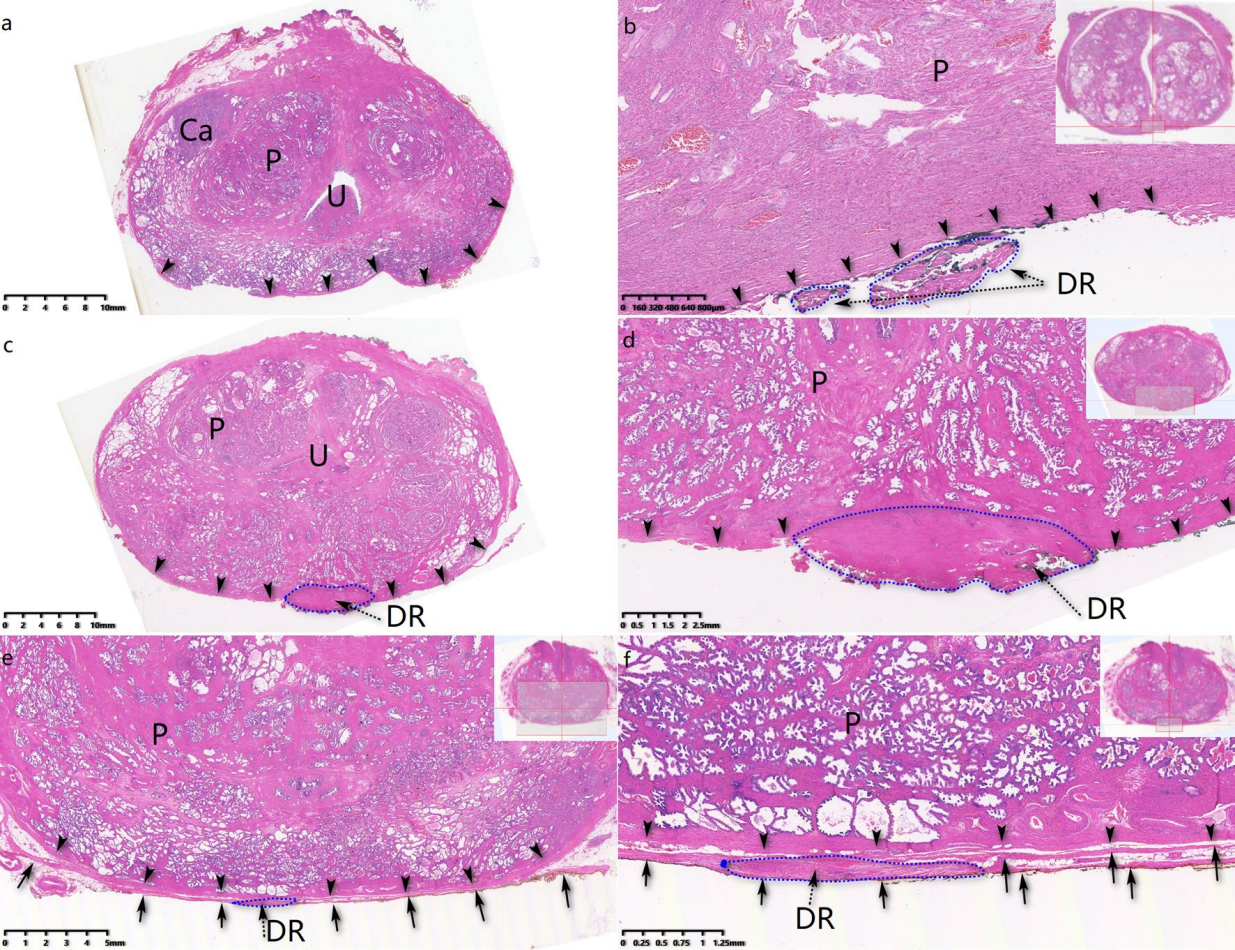
Different grades of DFS in RP, stained by hematoxylin and eosin. **a** In Grade-1 DF sparing procedure, neither DF nor DR was cut with prostate. **b** In Grade-2 DFS procedure, DF was preserved while DR fragments removed with prostate. **c**, **d** In Grade-2 DFS procedure, DF spared successfully while DR was totally cut away. **e**–**f** Grade-3 DFS was defined as neither DF nor DR spared. Black arrow head: prostate capsule; Ca: prostate cancer; DR: dorsal raphe (show in blue dot line circle); P: prostate; U: urethra. Black arrow: DF; Black arrow head: PC

DF has been demonstrated to be a multiple-layer structure both in cadaveric studies [15, 16] and histologic studies [17, 18] on specimen of RP. At the central-post direction of prostate and proximal of urethra, DF’s fascicles tend to fuse and adhere with PC to form a dorsal raphe (DR) [16, 18]. The tendinous DR, which extends distally to prostate apex and ends at perineal tendon, may act as a fulcrum to support prostate and proximal urethra [19]. At the lateral-post direction of prostate, DF disperses to connect with LAF (Fig. 2). Dalpiaz et al. [19] described DF as part of musculofascial suspension system, stabilizing prostate apex and proximal urethra. Interestingly, there was similar view on stress urinary incontinence (SUI) of females. DeLancey [20] delineated endopelvic fascia and anterior vaginal wall as the hammock-like supportive layer of urethra, which could stabilize and help to close urethra during cough. Sling, placing a supportive material behind urethra, had been a staple procedure for SUI and withstood the test of time [21]. Back to continence after RP, we hypothesized “hammock theory” still work on and preferred DF to be the critical structure (Figs. 1, 2 and 4), which could uplift vesicourethral anastomosis. Meanwhile, DR just acted as the fulcrum beneath urethra and contributed to closure of it when abdomen pressure increased. Hence, together with DR (also part of DF), DF was supposed to be the critical landmark for continence post RP. Our research also proved that DFS could significantly improve ImC (*OR* = 26.4, *P* < 0.01). However, because of individual difference, DF or DR could not be kept intact in every operation. So, we proposed a grading system for DFS procedure and our study showed that different grades can indicate different continence prognosis after RP (Fig. 4).

Considering that the scattering neurovascular plexus, which innervates corpora cavernosa and sphincter complex, is embedded in the multiple-layers of DF [3, 22], there was a minority of nerve fibers located in front of DF which would be dissected inevitably [23], while most of them are located in post-lateral and post regions of prostate [3] (Fig. 1). Tewari et al. [24] proposed a nerve-sparing (NS) grading system based on “landmark vein (LV)” lateral to prostate. An intra-fascial dissection was defined as grade-1, while an extra-fascial dissection was defined as grade-4. Srivastava et al. [25] demonstrated that grade-1 achieved more early continence than grade-4 (71.8% *vs* 43.5%), which may account for incomplete continence situation with intact DF.

DF-sparing technique doesn’t equal to complete periprostatic anatomy preservation or intra-fascial dissection. DFS procedure emphasizes dissecting before anterior layer of DF to protect the whole DF fascicles and the majority of neurovascular fibers, which is a procedure with a specific target to be spared, making the incision area selective but thorough. Whereas complete peri-prostate sparing technique aims to preserve functional structure as much as possible [14]. However, good functional recovery may sacrifice safety of tumor control. DF-targeted sparing procedure provides a compromised solution to the dilemma of urinary- or tumor-control.

With the same aim at preserving continence, Retzius-sparing (RS) technique puts more emphasis on preserving the structure in front of prostate [26]. RS was supposed to suspend and stabilize the bladder and anastomosis which might contribute to fast recovery of continence [26]. Compared with standard RP, lots of researches reported Retzius-sparing RP (RSRP) with better continence in short term, especially ImC [27, 28]. Given that more neurovascular plexus located post-lateral direction [3, 23], DFS might result in better continence from another perspective theoretically, but more prospective randomized controlled studies were needed to clarify that.

In our research, Group DFS achieved 83.3% ImC, 90.3% continence in 3 months, 91.7% in 6 months, 93.1% in 12 months (Fig. 3), better than Group Control at every time point with no difference in PSM (20.8% *vs* 20.7%, *P* = 1.0), especially no difference of PSM in post-lateral direction of prostate (4.2% *vs* 2.4%, *P* = 0.7).

Another key point to protect neurovascular plexus is the limited usage of energy devices, such as ultrasonic or electronic scalpels, bipolar coagulator, especially during prostate apex anatomy [29], in which nerve fibers penetrate DF or periprostatic tissue to innervate urethral sphincter. The electrical and thermal conduction may cause extra damage to neurovascular fibers nearby. Thereby, “blunt”, or “cold” dissection for prostate apex with finger, forceps, or scissors is preferred. The NS grading system proposed by Patel et al. [30] proved the idea. In Patel’s system, grade 5 NS was performed between “landmark artery (LA)” lateral to prostate and PC with neither sharp dissection nor energy devices, which resulted in complete nerve sparing (> 95%). Grade 4 NS (75%) was also performed between LA and PC with sharp dissection but without energy devices.

Last but not least, we preferred to pay more attention to ImC post RP. Temml et al. [31] testified that QoL wasn’t related to duration of incontinence but frequency and degree of incontinence, need for pads, etc. Coyne et al. [32] pointed out remarkable prevalence of 19.1% anxiety and 6.6% depression in patients with urinary incontinence (UI). Anxiety may trigger and aggravate UI in return. So better ImC may guarantee not only “extra” better continence in the future but also positive mental health.

Indeed, there are limitations in present research including small sample size, retrospective, and lack of randomization. Therefore, a prospective randomized controlled study with larger sample size should be organized for further verification.

## Conclusion

Denonvilliers’ fascia was supposed to act as the fulcrum and hammock for urinary control after RP. We recommend to preserve DF, minimize utility of energy devices during dissecting, which could probably contribute to best continence after RP without increase of PSM.

## Data Availability

All data generated or analyzed during this study are included in this published article.

## Abbreviations

BMI: Body mass index
CAD: Coronary artery disease
COPD: Chronic obstruction pulmonary diseases
CVD: Cerebrovascular diseases
DM: Diabetes mellitus
DF: Denonvillier’s fascia
DFS: DF-sparing
DR: Dorsal raphe
DVC: Dorsal vascular complex
HBP: High blood pressure
HE: Hematoxylin and eosin
IFT: Inter fascial tissue
IIEF-5: International Index of Erectile Function-5
ImC: Immediate continence
LAF: Levator ani fascia
LND: Lymph node dissection
LRP: Laparoscopic radical prostatectomy
Mmi-RRP: Mini-incision retropubic radical prostatectomy
NVB: Neurovascular bundle
ORP: Open radical prostatectomy
PC: Prostate capsule
PSM: Positive surgical margin
RARP: Robotic-assisted radical prostatectomy
RP: Radical prostatectomy
RS: Retzius sparing
RSRP: Retzius sparing radical prostatectomy
TURP: Transurethral resection of prostate
SUI: Stress urinary incontinence
SV: Seminal vesicles
VD: Vas deferens
