# Correction to: Denonvilliers’ fascia acts as the fulcrum and hammock for continence after radical prostatectomy

**DOI:** 10.1186/s12894-022-00968-y

**Published:** 2022-03-07

**Authors:** Xuwei Lu, Chang He, Sihong Zhang, Fan Yang, Zhuifeng Guo, Jiaqi Huang, Minke He, Jiawen Wu, Xia Sheng, Wenyao Lin, Jie Cheng, Jianming Guo, Hang Wang

**Affiliations:** 1 Department of Urology, Minhang Hospital, Fudan University, Shanghai, 201199 China; 2 Department of Urology, Zhongshan Hospital, Fudan University, Fenglin Rd 130, Shanghai, 200032 China; 3 Department of Pathology, Minhang Hospital, Fudan University, Shanghai, 201199 China; 4 Department of Urology, Xuhui Hospital, Fudan University, Shanghai, 200031 China

## Correction to: BMC Urology (2021) 21:176 https://doi.org/10.1186/s12894-021-00943-z

Following publication of the original article [1], the author reported that the equal contribution information was omitted. It has been presented in this correction article.
